# Respiratory Burst Oxidase Homolog D as a Modulating Component of Oxidative Response under Ammonium Toxicity

**DOI:** 10.3390/antiox11040703

**Published:** 2022-04-02

**Authors:** Maria Burian, Anna Podgórska, Monika Ostaszewska-Bugajska, Bożena Szal

**Affiliations:** Department of Plant Bioenergetics, Institute of Experimental Plant Biology and Biotechnology, Faculty of Biology, University of Warsaw, I. Miecznikowa 1, 02-096 Warsaw, Poland; mburian@biol.uw.edu.pl (M.B.); apodgorski@biol.uw.edu.pl (A.P.); m.ostaszewska@biol.uw.edu.pl (M.O.-B.)

**Keywords:** ammonium syndrome, apoplastic reactive oxygen species metabolism, respiratory burst oxidase homolog, growth retardation, apoplastic antioxidants

## Abstract

Delayed growth, a visible phenotypic component of the so-called ammonium syndrome, occurs when ammonium is the sole inorganic nitrogen source. Previously, we have shown that modification of apoplastic reactive oxygen species (apROS) metabolism is a key factor contributing to plant growth retardation under ammonium nutrition. Here, we further analyzed the changes in apROS metabolism in transgenic plants with disruption of the D isoform of the respiratory burst oxidase homolog (RBOH) that is responsible for apROS production. Ammonium-grown *Arabidopsis* *rbohd* plants are characterized by up to 50% lower contents of apoplastic superoxide and hydrogen peroxide. apROS sensing markers such as OZF1 and AIR12 were downregulated, and the ROS-responsive signaling pathway, including MPK3, was also downregulated in *rbohd* plants cultivated using ammonium as the sole nitrogen source. Additionally, the expression of the cell-wall-integrity marker FER and peroxidases 33 and 34 was decreased. These modifications may contribute to phenomenon wherein ammonium inhibited the growth of transgenic plants to a greater extent than that of wild-type plants. Overall, this study indicated that due to disruption of apROS metabolism, *rbohd* plants cannot adjust to ammonium toxicity and are more sensitive to these conditions.

## 1. Introduction

Like all other oxygenic organisms, plants need oxygen for primary metabolism; e.g., during respiration for cellular energy provision. In contrast to heterotrophs, they can produce and release oxygen during photosynthesis or absorb atmospheric oxygen. Oxygen dissolves in plant cells and is present in all compartments and the apoplast. However, oxygenic metabolism can produce toxic reactive oxygen species (ROS). ROS are active redox compounds and are considered important signaling molecules that reflect the changes inside or outside the cells [1,2,3,4,5,6]. Therefore, ROS can be involved in defense responses, regulation of plant metabolism, and growth processes [7,8,9]. A few enzymes involved in plant metabolism release ROS as a byproduct. However, not many enzymes are implicated in the active production of ROS [10], a process that has been mainly attributed to nicotinamide adenine dinucleotide phosphate (NADPH) oxidases, also known as respiratory burst oxidase homologs (RBOHs). These integral plasma membrane flavoproteins use cytosolic NADPH as the electron donor [11], and electrons transported across the membrane reduce ambient oxygen to release superoxide anions (O_2_^−^) into apoplastic space. RBOH activity is precisely regulated, e.g., by binding Ca^2+^ [12,13] or protein phosphorylation [14,15]. Most of the activation sites of RBOHs lie on the cytosolic side; therefore, they can be considered redox shuttles. Due to this transmembrane localization, the unique function of RBOHs can be related to the transmission of signals between the symplast and apoplast.

RBOHs cannot function alone in increasing ROS accumulation in the apoplast. As superoxide is a charged form of ROS, it cannot cross the plasma membrane and is highly reactive; therefore, it does not travel long distances. Superoxide may be converted nonenzymatically or enzymatically to hydrogen peroxide (H_2_O_2_) by superoxide dismutase (SOD). Hydrogen peroxide is a long-living and thus a more mobile form of ROS. In the apoplastic space, class III peroxidases (POXs) or amine oxidases can also lead to H_2_O_2_ production. POX activity is complex and depends on substrate availability, but the oxidative activity of POXs is considered a substantial part of extracellular ROS in response to pathogen infection [16]. Conversely, POXs can also be considered a ROS-scavenging enzyme, since POXs use H_2_O_2_ as a substrate to oxidize diverse compounds such as lignin in the peroxidative cycle. In addition, low-mass antioxidants, such as ascorbate and glutathione, together with ascorbate–glutathione cycle enzymes secreted into the apoplast, affect the scavenging capacity in this space [10,17]. However, the apoplastic antioxidant capacity is generally relatively low, so that the H_2_O_2_ wave can propagate easily. The accumulation of ROS in the apoplast can be perceived by receptor-like kinases (RLK) and activate mitogen-activated protein kinases (MAPK) [18], but signaling cascades are still not fully discovered. To create an ROS burst, different isoforms of ROS-metabolizing enzymes can transfer the information to cytosolic signaling.

RBOHs belong to a multigene family, containing 10 different members in the model plant *Arabidopsis thaliana* (RBOHA-J) [11]. The activation factors and tissue distribution of the different members of the family are slightly different [19,20,21]. The major isoforms are RBOHD and RBOHF, which are expressed in all plant organs and are mainly abundant in roots, followed by leaves, stems, inflorescences, and guard cells. These two isoenzymes seem to act synergistically in response to diverse stress factors [22,23]. RBOHD seems to have a higher ROS-producing capacity than RBOHF [15]. RBOHD is upregulated during exposure to diverse abiotic stress factors. Considering this, RBOHD contributes to acclimation to wounding, heat shock, cold temperature, heavy-metal toxicity, high light, salinity, osmotic stress, pathogen attack, hypoxia conditions, nitrogen deficiency, etc. ([20,21,24,25] and references therein). Currently, extensive research on the activation of RBOHF [26] is not available. RBOHF is mainly biotic-stress-inducible and participates in the hypersensitivity response [27]. Another function of RBOHF is regulating stomatal closure [28]. Other RBOHs are expected to have a more specialized function within the plant body and contribute to ROS accumulation during stress. The physiological activities of RBOH are still being explored, but the current knowledge is expected to be mainly related to stress perception and signaling [20]. Plants need to perceive all unfavorable conditions to adjust their metabolisms to the current stressful situation. Nutrient availability in the soil is an important environmental factor because it directly affects the plant’s nutritional status. Nitrogen (N) is one of the most important macronutrients for plant growth; hence, crop productivity is directly dependent on nitrogen availability. Plants can utilize nitrate (NO_3_^−^) or ammonium (NH_4_⁺) ions, but the uptake in this form affects specifically their metabolism; therefore, plants must recognize and distinguish between these nitrogen sources quickly. The drastic discrepancy between these two nitrogen forms can be reflected in plant development; for example, plant growth is inhibited in the presence of NH_4_⁺ as a sole nitrogen source. These toxicity symptoms of sole ammonium nutrition are called ammonium syndrome. However, despite studies on the physiological changes during NH_4_⁺ application (including N uptake carriers, assimilation enzymes, their regulators, and biochemical alterations), the cause of plant growth retardation remains unknown. It is reasonable to expect that the plant’s source of nitrogen directly affects its cellular redox metabolism. Assuming that the assimilation of nitrogen accounts for the consumption of 25% of photosynthetically generated reductants [29,30], then omitting nitrate reductase (NR) and nitrite reductase (NiR) activity under ammonium conditions leads to accumulation of reducing power that is available for cell functioning and can be used by other metabolic pathways/enzymes. Consequently, the over-reduction in ammonium-grown plants leads to ROS production [31,32] and possibly activation of redox-dissipating systems. Enzymes oxidizing NAD(P)H present in the cytosol might be, e.g., glutathione reductases, mitochondrial external NAD(P)H dehydrogenases, or possibly RBOHs [33,34].

It is worth considering the effects upstream of the intracellular metabolic performance of plants, including stress perception and transmission. As RBOHs are well-known signaling hubs and are involved in stress responses, these enzymes are thought to contribute to the development of ammonium syndrome. Our previous study showed that during long-term NH_4_⁺ nutrition, RBOH activity was strongly upregulated, correlating with higher apoplastic ROS production in *Arabidopsis thaliana* [35]. To gain insight into the potential roles of RBOHs in nitrogen responses, we investigated *rbohd* mutants of *Arabidopsis thaliana*. We found that the dysfunction of RBOHD played a role in the progression of NH_4_⁺ toxicity. Understanding apoplastic ROS metabolism is a key goal when considering nitrogen signaling.

## 2. Materials and Methods

### 2.1. Plant Material and Growth Conditions

Seeds of *Arabidopsis* of two independent lines, SALK_109396C and SALK_035391C (named in this manuscript as *rbohd1* and *rbohd2*, respectively) insertional mutants with disrupted *RBOHD* expression, were purchased from the Nottingham *Arabidopsis* Stock Center (NASC). Homozygous mutants were isolated by PCR-based genotyping using the gene-specific primers. *Arabidopsis thaliana* ecotype Columbia-0-control (wild type, WT) and *rbohd1* and *2* mutants were grown in hydroponic culture using an Araponics growth system. Seeds were placed in holders on 0.5 × Murashige and Skoog [36] medium (MS, M5524, Merck; Darmstadt, Germany) with the addition of 1% (*w*/*v*) agar. Plants were grown in a growth chamber under short-day light conditions (8 h light/16 h dark cycle) under 150 μmol m^−2^ s^−1^ photosynthetically active radiation (PAR) and day/night temperatures of 21 °C/18 °C, as in [37]. After one week of germination in water, plants were supplemented with a full growth medium that was constantly aerated and replaced twice a week. The nutrient solution was composed of 1.5 mM KH_2_PO_4_, 2.5 mM KCl, 0.7 mM CaSO_4_·2H_2_O, 0.8 mM MgSO_4_·7H_2_O, 0.06 mM NaFe-EDTA, 5 mM CaCO_3_, and micronutrient solution (0.28 μM CuSO_4_·5H_2_O, 0.4 μM ZnSO_4_·7H_2_O, 0.15 μM KI, 0.20 μM KBr, 0.20 μM Na_2_MoO_4_·2H_2_O, Merck), and 2.5 mM Ca(NO_3_)_2_·4H_2_O or 2.5 mM (NH_4_)_2_SO_4_ (Chempur; Piekary Śląskie, Poland) as the nitrogen source. After eight weeks of hydroponic culture, plant material was collected (whole leaves) in the middle of the photoperiod for further experiments. Randomly selected rosettes from two independent nitrate or ammonium plant cultures were weighed for fresh weight (FW) determination.

### 2.2. Phosphorylated Pyridine Nucleotide Estimations

NADP(H) was extracted from leaf samples as described by [38] with modifications. A total of 50 mg of frozen tissue was homogenized briefly in 400 µL of 0.1 M HCl (for NADP extraction) or 0.1 M KOH (for NADPH extraction) in 50% (*v*/*v*) ethanol using a mixer mill. Reduced and oxidized pyridine nucleotides were determined using an enzymatic cycling method as described in [39].

### 2.3. Isolating Extracellular Fluid

Extracellular washing fluid (EWF) was isolated from fresh leaves by vacuum infiltration, as described in [40]. Fluids were extracted using a centrifuge (Eppendorf 5804R centrifuge; Hamburg, Germany) and were directly filtered into 0.1 M HClO_4_ to stop the metabolic degradation of molecules. The samples were maintained at 4 °C until analysis.

### 2.4. Isolating Cell-Wall Protein Fraction and Estimation of Peroxidase Activity

Proteins from cell walls were extracted according to the method described in [41]. Frozen leaf samples were homogenized in 0.05 M HEPES-KOH, pH 6.5, and the homogenates were centrifuged at 5000× *g* (Eppendorf 5804R centrifuge) for 10 min. The recovered pellet was washed thrice with cold water. Proteins from the crude cell-wall samples were extracted using 0.05 M HEPES, 1 M NaCl, and protease inhibitor cocktail (cOmplete Ultra Mini EDTA-free Easy pack, 05892791001, Merck) under continuous agitation at 4 °C for 12 h. Homogenates were centrifuged at 13,000× *g* for 15 min. Extracts were desalted and concentrated on filter columns according to the manufacturer’s protocol (Microcon-10, MRCPRT010; Merck), and the obtained samples were used for the POX activity assay. POX activity was measured by monitoring the oxidation of guaiacol (G5502, Merck), as described in [42].

### 2.5. Visualization of Reactive Oxygen Species Level in Leaf Tissue

A method for quick in vivo visualization of superoxide radicals in plant leaves was adapted from [43]. Leaves were infiltrated with 0.1% nitroblue tetrazolium (NBT, N6876, Merck) solution using a vacuum pump for several minutes. Simultaneously, the NBT staining was performed by adding 8 µM diphenylene iodonium chloride (DPI, D5767, Merck), a commonly used RBOH inhibitor. The differences in NBT-dependent blue staining of leaves in the presence and absence of DPI were representative of RBOH activity producing superoxide. Coloration by NBT staining in control plants was presented as a value of 1. The staining intensity of NBT on leaves was quantified using Image Processing and Analysis in Java software (ImageJ, U. S. National Institutes of Health, Bethesda, MD, USA) using the method described in [44].

### 2.6. Determining Metabolites Related to ROS Metabolism

The hydrogen peroxide concentration was measured using the fluorescence method by applying homovanillic acid (HVA, H1252, Merck), following [45]. The H_2_O_2_ content in the foliar extracts and EWF fractions was determined in the presence of 500 µM HVA and 10 U of horseradish peroxidase (HRP, P6782, Merck). Data were collected at λ = 315 nm and 425 nm for the excitation and emission wavelengths, respectively. The H_2_O_2_ concentration was estimated using an internal standard, which was created by adding 1 nmol H_2_O_2_ to the reaction mixture of each sample. Quantification of lipid peroxidation in leaf tissues was performed according to [46] as the level of the specific reaction product malondialdehyde (MDA). MDA content was measured in the presence of thiobarbituric acid (TBA, T5500, Merck), and simultaneous control reactions were performed without the addition of TBA. The absorbance of each sample was measured at λ = 440 nm, 532 nm, and 600 nm to correct nonspecific product contamination. The ascorbate content was measured in leaf extracts and EWF using a colorimetric assay according to [47] with modifications [48]. The potential of reduced ascorbate (AsA) to reduce ferric ions was used to estimate α-α-bipyridyl (D216305, Merck)-dependent color development at λ = 525 nm. The total ascorbate pool was estimated by dithiothreitol (DTT, D0632, Merck) reduction of dehydroascorbate (DHA) in each sample. DHA was calculated after separating the absorbance of the reduced form from the total ascorbate pool. Glutathione content was measured in leaf extracts using the enzymatic cycling method described in [49]. For the assay for glutathione disulfide (GSSG), 2-vinyl pyridine (132292, Merck) was added to each sample to mask reduced glutathione (GSH). The measurement of GSH was based on the reduction of 5,5′-dithiobis-2-nitrobenzoic acid (D8130, Merck) and recording the absorbance at λ = 412 nm. 

### 2.7. Plasma Membrane Isolation

Right-side-out vesicles of the plasma membrane were isolated using a two-phase system composed of dextran 500 (6.4%; 92192, Carl Roth; Karlsruhe, Germany) and polyethylene glycol (6.4%; PEG, 305413, Merck) supplemented with 5 mM KCl, as described in [50].

### 2.8. Immunoblotting Analyses

Isolated plasma membrane were subjected to sodium dodecyl sulfate-polyacrylamide gel electrophoresis (SDS-PAGE) to determine RBOHD protein abundance. Protein content in the samples was determined using the *RC DC*^TM^ Protein Assay kit (5000121, Bio-Rad Laboratories, Hercules, CA, USA). Protein samples (10 µg protein from the plasma membrane sample per lane) were separated using SDS-PAGE (10% polyacrylamide resolving gel) according to a standard protocol. The polypeptides were electroblotted onto a nitrocellulose membrane and probed overnight at 4 °C with a primary antibody anti-RBOHD (AS15 2962) diluted 1:1000; these were all purchased from Agrisera, Vännäs, Sweden. Antirabbit antibodies conjugated to HRP (diluted 1:25,000; 170615, Bio-Rad) were used as a secondary antibody. Visualization was performed using a chemiluminescent reagent system, and the corresponding stained enzyme isoforms were identified based on their molecular masses. After correcting for background, the specific protein levels were quantified based on the densitometry of bands using Image-Lab 5.2 software (Bio-Rad). The results were expressed in relation to WT plants grown in nitrate (value of 1). The carbonylated protein derivatives separated by SDS-PAGE were quantified according to the method in [51]. Antibodies to the dinitrophenyl group (diluted 1:1000; D9656, Merck) were used as primary antibodies. The amounts of oxidized proteins were visualized through chemiluminescence, and the staining intensity of the entire blot lane was quantified through densitometry using Image-Lab 5.2 software (Bio-Rad) after background correction.

### 2.9. Quantitative RT-PCR Analyses

RNA isolation, complementary DNA generation, and RNAse digestion were conducted as described in [52]. RT-qPCR reactions were measured in iTaq universal supermix (1725121, Bio-Rad), carried out at 60 ˚C in a thermocycler (CFX-Connect, Bio-Rad). Transcript abundance was normalized to the transcript level of the reference gene protein phosphatase 2a (*PP2A*) according to [53]. The results were expressed in relation to the WT plants grown in nitrate (value of 1), according to the method in [54]. The list of primers used to measure the transcript abundance of auxin-induced in root cultures 12 (*AIR12*, AT3G07390), feronia (*FER*, AT3G51550), *GR1* (AT3G24170), *GR2* (AT3G54660), mitogen-activated protein kinase 3 (*MPK3*, AT3G45640), oxidation-related zinc finger 1 (*OZF1*, AT2G19810), *POX33* (AT3G49110), *POX34* (AT3G49120), and cell wall-associated kinase 1 (*WAK1*, AT1G21250) is shown in Supplementary Materials Table S1.

### 2.10. Determining Degree of Pectin Esterification

Alcohol insoluble residue (AIR) was isolated with the method described in [55] with modifications [56]. Pectin from AIR was extracted as described in [57]. The levels of pectic unesterified homogalacturonan (HG) and highly methyl esterified HG were determined using enzyme-linked immunosorbent assay (ELISA), as described in [56] using LM19 and LM20 antibodies (Plant Probes, Inc., Leeds, United Kingdom; diluted 1:10). Antibody binding was quantified using a 3,3,5,5-tetramethylbenzidine liquid substrate system (T0440, Merck) according to the manufacturer’s protocol.

### 2.11. Statistical Analysis

Results are expressed as the mean value ± standard deviation (SD) of n measurements (*n* = 3–25) taken from at least two independent plant cultures. In our previous studies [17,35,58], we described and widely discussed differences between nitrate- and ammonium-grown WT plants, and we now indicate only the differences resulting from RBOHD dysfunction. To analyze the statistical significance of observed differences between WT and *rbohd* plants grown either in nitrate or ammonium, a one-way analysis of variance (ANOVA) with Tukey’s post hoc test was performed using Statistica 13.3 software (StatSoft, Inc., Tulsa, OK, USA). The differences between WT and *rbohd* mutants grown in nitrate or ammonium are indicated with asterisks (*p* ≤ 0.05 *; *p* ≤ 0.01 **). Only changes observed in both *rbohd* genotypes (the same trend in *rbohd1* and *rbohd2*) plants were assumed to be linked to RBOHD disruption and are discussed. 

## 3. Results

### 3.1. The Influence of Deficiency in RBOHD on Plant Phenotype under Different Nitrogen Conditions

**Figure 1:**
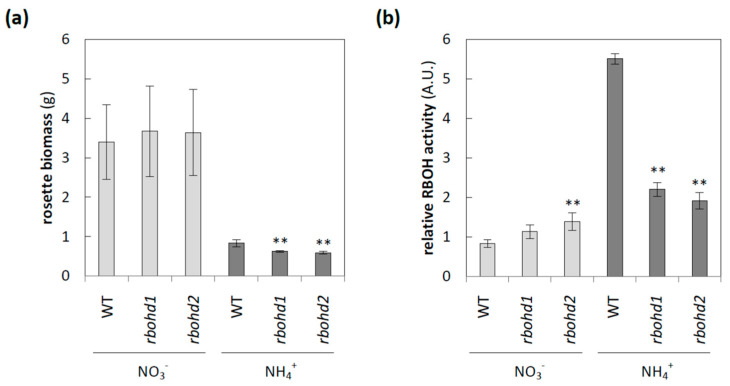
Phenotypic characterization of *rbohd* plants. (**a**) Rosette biomass of 8-week-old WT and *rbohd* plants grown in nitrate or ammonium as the sole nitrogen source. (**b**) Relative activity of RBOH measured as DPI-sensitive superoxide production. Comparison tests between *rbohd* and WT plants were performed on the mean of experimental conditions for the nitrate (light grey) or ammonium (dark grey) growth regime separately. Statistical differences calculated using one-way analysis of variance (ANOVA) with Tukey’s post hoc test are indicated at *p*-value ≤ 0.01 **.

The transcript level of RBOHD, which is believed to be the dominant isoform of RBOH in *Arabidopsis* leaves [15], decreased in response to long-term ammonium nutrition [35]. Surprisingly, when the activities of RBOHs and RBOHD protein levels were analyzed, an increase in both these parameters was observed in WT plants due to cultivation in the presence of ammonium [35] (Figure 1b; Supplementary Materials Figure S1). Therefore, we decided to analyze the role of RBOHD under different nitrogen nutrition conditions in detail. The rosette size of both *rbohd* genotypes had biomass comparable to that of WT plants during nitrate nourishing, but eight-week cultivation in ammonium led to growth stunting of *rbohd* plants compared to WT plants (Figure 1a). In order to understand the effect of reduced nitrogen form availability for *rbohd* functioning, we analyzed the phosphorylated nucleotide redox pool, since it was the substrate for RBOH activity, but no significant changes were observed between nitrogen growth regimes (Supplementary Materials Figure S2).

### 3.2. Changes in ROS Metabolism

**Figure 2:**
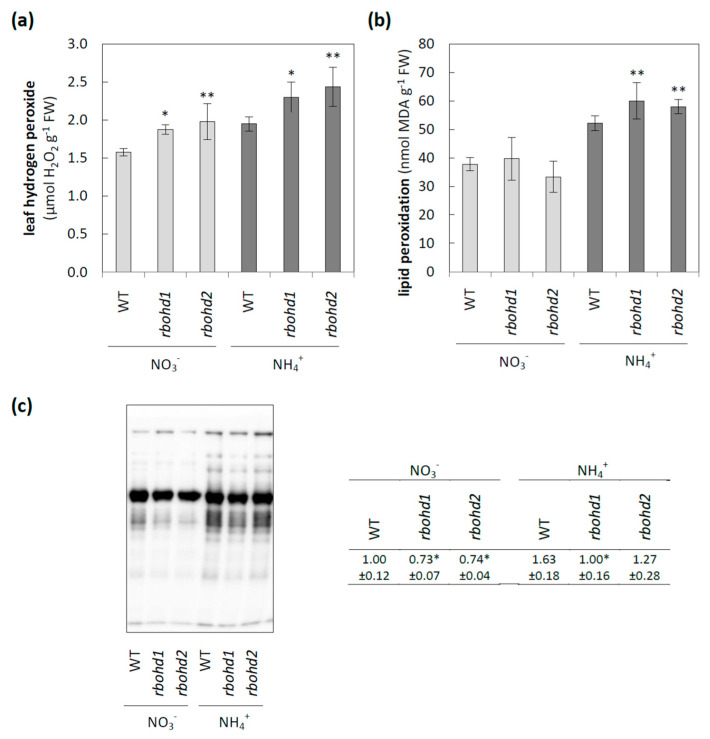
ROS level and biomolecule injuries in leaf tissue of WT and *rbohd* plants grown in nitrate or ammonium as a nitrogen source. (**a**) Leaf hydrogen peroxide concentration. (**b**) Membrane injury is measured as malondialdehyde content. (**c**) The profile of protein carbonylation in leaf tissues. Comparison tests between *rbohd* and WT plants were performed on the mean experimental conditions for the nitrate (light grey) or ammonium (dark grey) growth regime separately. Statistical differences calculated using one-way analysis of variance (ANOVA) with Tukey’s post hoc test are indicated at *p*-value ≤ 0.01 ** and *p*-value ≤ 0.05 *.

In our previous work [37], we focused on WT plants and found that ammonium supply led to oxidative stress in leaf tissue of WT plants as manifested by, among other things, an increase in hydrogen peroxide content, higher lipid peroxidation, and increased protein carbonylation. Continuing the research on how changes in redox homeostasis affect cellular functioning, here we analyzed the metabolic consequences of the RBOHD dysfunction when plants were grown in ammonium conditions. The loss of RBOHD mostly resulted in increased hydrogen peroxide content under both growth treatments (Figure 2a), but ROS levels were the highest in ammonium conditions. In addition, ammonium nutrition further increased oxidative injuries of lipids (Figure 2b), but not proteins, in *rbohd* plants (Figure 2c).

**Figure 3:**
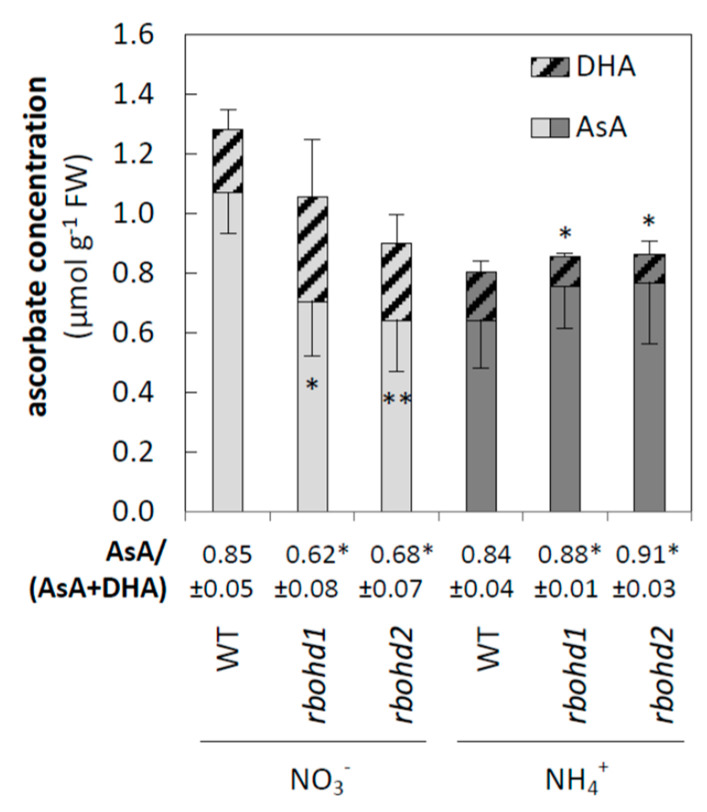
Ascorbate and dehydroascorbate concentration in leaves of WT and *rbohd* plants grown in medium containing nitrogen in the form of nitrate or ammonium ions. Comparison tests between *rbohd* and WT plants were performed on the mean experimental conditions for the nitrate (light grey) or ammonium (dark grey) growth regime separately. Statistical differences calculated using one-way analysis of variance (ANOVA) with Tukey’s post hoc test are indicated at *p*-value ≤ 0.01 ** and *p*-value ≤ 0.05 *.

In order to connect toxicity symptoms of ammonium-grown *rbohd* with the metabolites responsible for redox buffering, low-mass antioxidant systems were also analyzed. A lowered AsA/DHA ratio and upregulation of enzymes constituting the Foyer–Halliwell–Asada cycle indicated antioxidant activation by ammonium in WT plants, similar to the results described in [37]. Both genotypes of *rbohd* showed an increased level of ascorbate pool oxidation when grown in nitrate (Figure 3). As opposed to that, *rbohd* mutants increased the redox state of the ascorbate pool in response to ammonium cultivation compared to WT plants (Figure 3).

**Figure 4:**
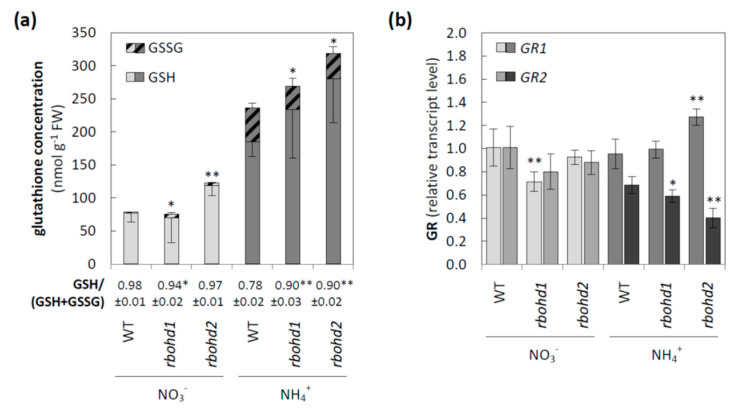
Glutathione-related leaf antioxidant system in WT and *rbohd* plants cultivated in the presence of nitrate or ammonium as a sole nitrogen source. (**a**) Reduced glutathione and glutathione disulfide concentration. (**b**) The transcript level of glutathione reductase 1 and glutathione reductase 2. Comparison tests between *rbohd* and WT plants were performed on the mean of experimental conditions for the nitrate (light grey) or ammonium (dark grey) growth regime separately. Statistical differences calculated using one-way analysis of variance (ANOVA) with Tukey’s post hoc test are indicated at *p*-value ≤ 0.01 ** and *p*-value ≤ 0.05 *.

*Rbohd* mutants have tended to increase the level of oxidized glutathione in response to nitrate, but the GSH/(GSH + GSSG) ratio was significantly changed only in *rbohd1* plants. However, similar to ascorbate, the glutathione pool was more reduced under ammonium nutrition in *rbohd* plants than in WT plants (Figure 4a). Since GR in the *Arabidopsis* genome is encoded by two isoforms with different intracellular localizations, the transcript levels of both genes were checked. No clear trend was observed for GR1, since the studied lines of transformants showed different regulations of this gene in response to nitrogen source; nitrate nutrition resulted in a lowered expression of *GR1* in *rbohd1*, whereas ammonium nutrition increased the expression of *GR1* in the *rbohd2* line. Simultaneously, the expression of *GR2*, downregulated by ammonium in WT plants in both transformants, was further repressed under ammonium cultivation (Figure 4b).

### 3.3. Modification of Apoplastic ROS Metabolism and Pectic Glycan Level Esterification

**Figure 5:**
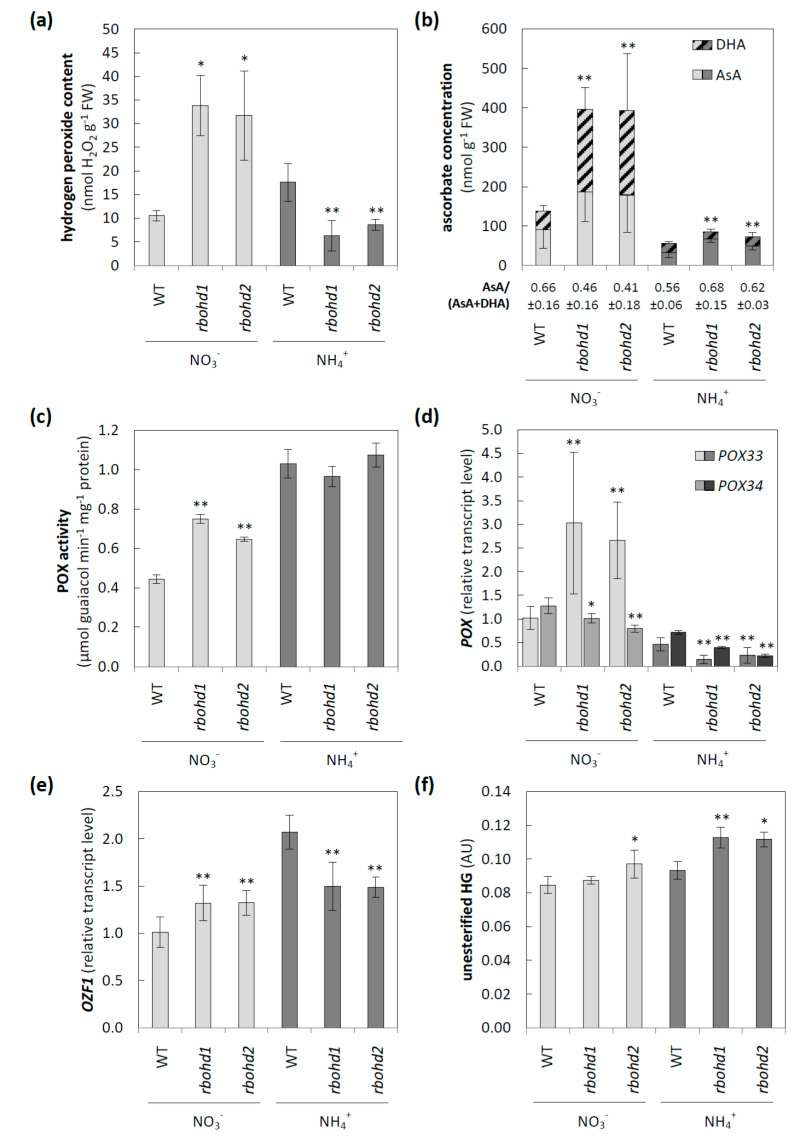
Changes in apoplastic metabolism of reactive oxygen species and the level of esterification of pectin resulting from RBOHD dysfunction in *Arabidopsis* grown in nitrate or ammonium as a sole nitrogen source. (**a**) The level of hydrogen peroxide in extracellular space. (**b**) The concentration of ascorbate and dehydroascorbate in leaf extracellular washing fluid. (**c**) Peroxidase activity in cell-wall-associated proteins. The transcript levels of (**d**) peroxidase 33 and peroxidase 34 and (**e**) oxidation-related zinc finger 1. (**f**) The level of unesterified pectin isolated from alcohol insoluble residue. Comparison tests between *rbohd* and WT plants were performed on the mean experimental conditions for the nitrate (light grey) or ammonium (dark grey) growth regime separately. Statistical differences calculated using one-way analysis of variance (ANOVA) with Tukey’s post hoc test are indicated at *p*-value ≤ 0.01 ** and *p*-value ≤ 0.05 *.

Since separated pools represented the cytosolic and extracellular ROS levels, and RBOH activity directly affected the apoplast, we focused on ROS metabolism in this space. First, an increase in hydrogen peroxide level under nitrate and a decrease under ammonium compared to WT plants was observed in both *rbohd* transformants (Figure 5a). In line with these results, the apoplastic pool of DHA in *rbohd* plants was increased or decreased under nitrate or ammonium, respectively. Additionally, impairment of RBOHD activity seemingly led to more than twice the pool of apoplastic ascorbate when the plants were cultivated in nitrate (Figure 5b). AO activity, which may influence the apoplastic ascorbate redox state, was unaffected by long-term ammonium nutrition (results not presented). Another ROS source in the apoplast may be POXs; therefore, we checked its contribution to apoplastic ROS content. Cell-wall-associated POX activity was enhanced in *rbohd* transformants compared to WT under nitrate conditions (Figure 5c). Ammonium nutrition led to an even higher increase in POX activity, but the differences between genotypes were not observed (Figure 5c). POXs are encoded by a multigene family, out of which the expressions of two genes, POX33 and 34, were analyzed. Under nitrate conditions, *POX33* was strongly upregulated, and *POX34* was slightly downregulated in *rbohd* transformants compared to control plants (Figure 5d). Ammonium nutrition led to a decrease in both *POX33* and *POX34* transcript levels in both *rbohd* plants as compared to WT plants (Figure 5d). Following the above-described changes, the transcript level of *OZF1*, which is believed to be a marker of oxidative stress in extracellular space, was enhanced or decreased in *rbohd* transformants under nitrate or ammonium conditions, respectively, compared to WT plants (Figure 5e).

Since pectin properties, including the level of HG esterification, may be factors activating the signaling pathways connected to the apoplast/cell-wall status, therefore in the next step, we checked whether any such changes were induced by RBOHD disruption under different nitrogen nutrition conditions. A significantly increased level of unesterified HG was observed in both *rbohd* plants in response to ammonium nutrition (Figure 5f). The highly methyl-esterified HG level was not modified either by RBOHD dysfunction or by nitrogen source (Supplementary Materials Figure S3).

### 3.4. Signaling Pathways Activated by Changes in Extracellular Space

**Figure 6:**
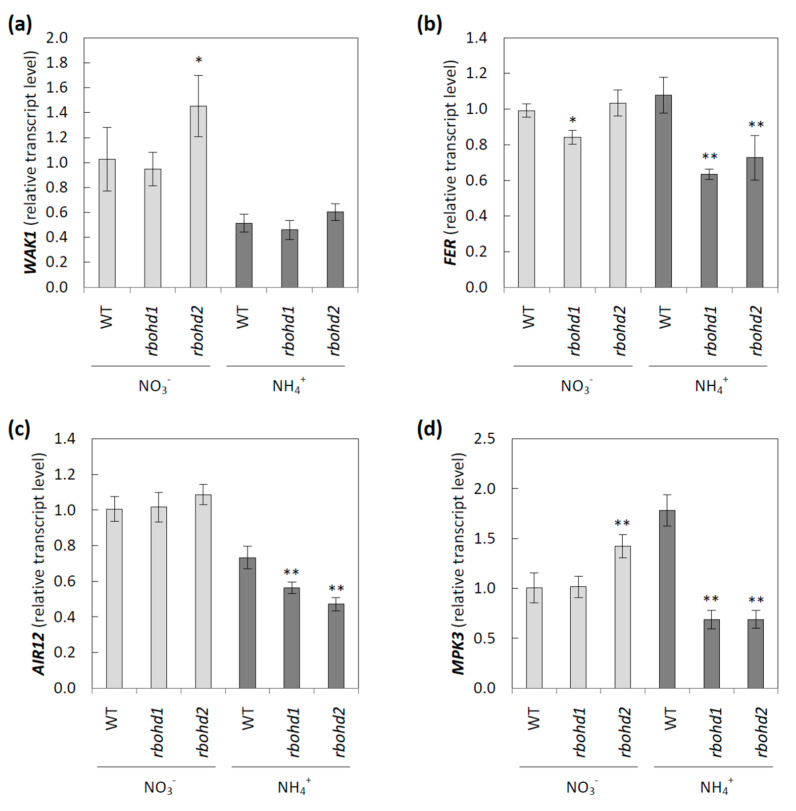
Signaling pathways related to cell-wall changes or to apoplastic reactive oxygen species metabolism in WT and *rbohd* plants grown in nitrate or ammonium as a sole nitrogen source. The transcript level of cell wall-associated kinase 1 (**a**), feronia (**b**), auxin-induced in root cultures 12 (**c**), and mitogen-activated protein kinase 3 (**d**). Comparison tests between *rbohd* and WT plants were performed on the mean experimental conditions for the nitrate (light grey) or ammonium (dark grey) growth regime separately. Statistical differences calculated using one-way analysis of variance (ANOVA) with Tukey’s post hoc test are indicated at *p*-value ≤ 0.01 ** and *p*-value ≤ 0.05 *.

A continuum between the symplast and apoplast is formed in plant tissues, with several metabolites being exchanged between these compartments. Intracellular metabolism can be modified by changes in cell-wall properties or an imbalance in the apoplastic redox state, including changes in extracellular ROS metabolism [58]. In the study of RBOHD function under ammonium conditions, we further focused on primary signaling routes dependent on changes in apoplastic metabolism. WAK1 is believed to be a sensor of pectin modification, and its expression was strongly downregulated by ammonium nutrition in all genotypes (Figure 6a). Additionally, expression of FER, which is also postulated to be a sensor of pectin [59], was lower in *rbohd* transformants than in WT plants under ammonium nutrition (Figure 6b). The expression of *AIR12* encoding monoheme cytochrome b, which may influence apoplast ROS status [60], was reduced in all tested genotypes by ammonium cultivation, but was further repressed in *rbohd* plants, as compared to WT plants (Figure 6c). Furthermore, the transcript level of one mitogen-activated protein kinase, *MPK3*, which is believed to be strongly associated with stress signaling [61], was analyzed. *MPK3* expression was upregulated in WT plants but downregulated in *rbohd* transformants in response to ammonium stress (Figure 6d).

## 4. Discussion

### 4.1. RBOHD Disruption Leads to Changes in Apoplast-to-Symplast Signaling under Ammonium Stress

Extracellular space is a source of signaling clues that must be received and converted to intracellular changes for producing a metabolic effect that allows cells to adapt to the stress factor. Such signaling may be dependent on changes in metabolites (such as ROS) or ion concentrations (e.g., calcium ions, [62]) or transmitted through specific transmembrane enzymes anchored in the extracellular space, such as wall-associated kinases (WAKs) [63].

MPK3 is one of the dozens of kinases included in the conserved mitogen-activated protein kinase (MAPK) cascade, allowing the transfer of signals from external-stimuli-activated sensors to target molecules. This allows the response to be triggered inside the cell [64]. MAPK activation is often observed in response to an increase in apoplastic ROS levels, enhancing antioxidant defense systems [65]. *Arabidopsis* MPK3 is a hydrogen-peroxide-induced MAPK [64]. Under ammonium nutrition, lowered apoplastic hydrogen peroxide (Figure 5a) and decreased MPK3 transcript levels (Figure 6d) were observed in *rbohd* plants compared to WT plants. However, we did not observe such a positive correlation in *rbohd* plants under nitrate treatment, in which apoplastic hydrogen peroxide was highly increased (Figure 5a), and no significant differences in MPK were detected (Figure 6d). However, in most studies, the interaction of MAPK, including MPK3 with an oxidative burst mediated by RBOHs, has been suggested [66]. Apoplastic hydrogen peroxide may be produced mainly by POXs in nitrate-grown *rbohd* plants (Figure 5c,d).

In addition, AIR12 transcript levels were downregulated in response to ammonium, and even to a higher degree, in ammonium-grown *rbohd* plants (Figure 6c). The role of AIR12 in plasma membrane–apoplast redox reactions in vivo remains unclear [60], but its involvement in redox signaling has been strongly indicated [67]. Since AIR12 is not a transmembrane protein, the interaction of AIR12 with other proteins is needed to perform such a role. AIR12 is a component of the complex redox system of lipid rafts in the plasma membrane [68,69]. It is noteworthy that the MAPK cascade and AIR12 protein link ROS and auxin signaling [70,71], which may be interesting in light of the recently shown disturbances in phytohormone metabolism in plants grown under ammonium stress [72,73].

RBOHD dysfunction also influenced the abundance of the FER transcript under ammonium nutrition (Figure 6b), which is believed to be a cell-wall-integrity mechanosensor [74]. It is dependent on pectin metabolism [75] and controls the extensibility of the cell wall [76]. FER was shown to interact with leucine-rich-repeat extensins (LRXs), and both components facilitate sensing of extracellular signals, including apoplast acidification and cell-wall loosening, and convey them to the symplast [77]. FER functioning is crucial for maintaining de-esterified pectin [78]. Therefore, the link between lower levels of FER transcripts (Figure 6b) and higher levels of de-esterified pectin in the cell walls of *rbohd* plants (Figure 5f) under ammonium nutrition is unclear.

Ammonium nutrition also changed the transcript level of WAK1 (Figure 6a), a receptor-like kinase whose extracellular domain binds to pectins [79,80]. The extracellular domains of WAKs preferentially bind to de-esterified pectins [81], and the cytoplasmic serine and threonine domain leads to the activation of genes required for cell expansion [63]. Previously, it was shown that a decrease in WAK protein arrested cell elongation, resulting in the dwarf phenotype [82,83]. Similarly, ammonium-grown plants, in which the WAK transcript level was lowered (Figure 6a), showed stunted growth (Figure 1a). Additionally, the engagement of MPK3 in the WAK-induced signaling pathway was shown [84,85], and following this observation, MPK3 was downregulated in *rbohd* genotypes grown under ammonium nutrition (Figure 6d). Extracellular hydrogen peroxide production and WAK expression were positively correlated [86], and a similar trend was observed in our experiment in *rbohd* plants, but not in WT plants (Figure 6a and Figure 5a).

**Figure 7:**
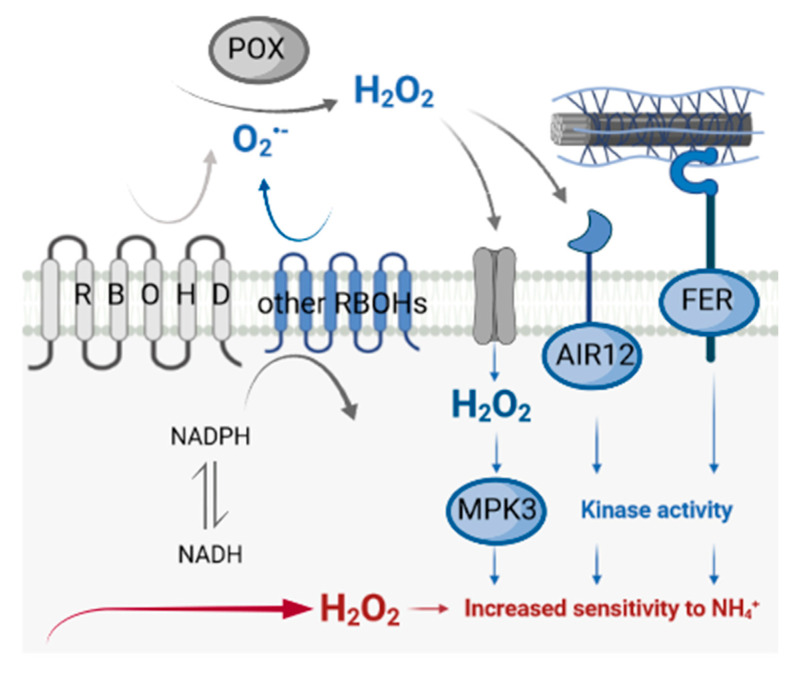
Metabolic effects of RBOHD disfunction for plants nourished with ammonium. When the major RBOHD is not active, other RBOHD isoforms cannot compensate for its activity, and O_2_^−^ production is diminished. Lower O_2_^−^ availability or POX activity can lead to decreased H_2_O_2_ contents as their product. As a consequence, H_2_O_2_-responsive receptors such as AIR12 are downregulated. Another cell-wall receptor is FER, and decreased pectin methyl-esterification leads to lower expression. Further signaling pathways from these receptors are not activated in cells, such as in the MAPK cascade. Since the H_2_O_2_ level is low in the apoplast, it would also pass to a minor extent into the cytosol; hence, direct activation of intracellular signaling kinases such as MPK3 is limited. Impaired apoplast/cytosol signaling does not induce defense/antioxidant systems, and therefore oxidative stress develops in cells. Due to these disturbances, rbohd showed high sensitivity to ammonium fertilization. Reduced responses are highlighted in blue, and red shows induced reactions. The scheme was created with BioRender.com.

Overall, RBOHD dysfunction affected all tested signaling elements in ammonium-grown plants. Therefore, our results indicated that RBOHD activity is an indispensable component of ROS- and cell-wall-dependent apoplast-to-symplast signaling (Figure 7).

### 4.2. Stunted Growth of Rbohd Plants under Ammonium Nutrition May Be Associated with Altered ROS Metabolism in the Apoplast and Modification of Cell-Wall Properties

Apoplastic ROS regulates plant growth and may inhibit development in response to different environmental stress conditions, including ammonium stress [35,58].

Our results suggested that under ammonium nutrition, other isoforms of RBOH were unable to compensate for RBOHD disruption, since RBOH activity was significantly decreased under ammonium nutrition in *rbohd* plants compared to WT plants (Figure 1b). In addition, under ammonium supply, activation of POX activity was not shown due to RBOHD dysfunction (Figure 5c,d); however, ammonium treatment increased POX activity in WT, similar to previously observed effects [35]. Our results also indicated changes in cell-wall composition due to RBOHD disruption. A higher level of de-esterified pectin was observed under ammonium nutrition (Figure 5f). The esterification level of homogalacturonan may be an important factor in determining cell-wall properties, since covalently crosslinked pectin contributes to the stiffness of the cell wall [87]. Additionally, pectin in the egg-box conformation possibly creates a matrix for POX docking [88,89]. Due to the higher level of de-esterified HG in *rbohd* plants under ammonium nutrition (Figure 5f), an increased pool of POXs may be associated with the solid phase of the extracellular space and affect plant growth.

Under nitrate nutrition in *rbohd* plants, H_2_O_2_ levels were enhanced in the apoplast (Figure 5a), together with a highly increased ascorbate pool, mainly due to a higher DHA level (Figure 5b). The changes in apoplastic ROS metabolism observed in transformants indicated that disruption of RBOHD activated some compensatory mechanism, leading to an increase in apoplast ROS production. An enhanced apoplastic hydrogen peroxide level was observed in the *rbohd* plants under control growth conditions (Figure 5a), which also was reported previously [90]. This confirmed our observation that the dysfunction of RBOHD activated other apoplastic ROS-producing systems. Since RBOHD was proposed to be activated by the presence of hydrogen peroxide [91], it may be assumed to be an amplifier, rather than an initial generator, of ROS burst in response to stress [92].

Our results indicated that POXs were activated in the *rbohd* plants under nitrate nutrition (Figure 5c,d), which may have increased the apoplastic H_2_O_2_ level. It has been proven that POX can form H_2_O_2_ directly without spontaneous or SOD-catalyzed dismutation of superoxide ions in the apoplastic space. POX activation is associated with pathogen-induced apoplastic ROS burst in several plant species [93,94,95]. Cell-wall POXs have a dual role (as ROS-source and ROS-detoxification mechanisms), and until now, unidirectional mechanisms among dozens of known individual POX isoforms encoded in the *Arabidopsis* genome have not been strictly proven. POX33 may play a major role in generating apoplastic H_2_O_2_ [96], and in the present study, POX33 was strongly upregulated in nitrate-grown *rbohd* plants (Figure 5e).

To summarize, lower apoplastic H_2_O_2_ levels (Figure 5a) were detected in *rbohd* plants than in WT plants under the ammonium regime. In line with the observed apoplastic changes in *rbohd* plants in ROS metabolism (Figure 5b,c), OZF1 transcript level was decreased under ammonium conditions in *rbohd* plants and slightly increased in nitrate-grown plants (Figure 5e). Transcripts of plasma-membrane-localized OZF1 were highly induced by treatment with hydrogen peroxide [97]; therefore, they may be recognized as a marker of extracellular oxidative stress.

### 4.3. Dysfunction of RBOHD Influences Intracellular Redox State of Cells

In all genotypes under ammonium nutrition in plant tissues, reduced pyridine nucleotides were in excess due to nitrogen metabolism, omitting the steps of nitrate and nitrite reduction. However, in *rbohd* plants, cytosolic NADPH was not used by the main RBOH isoform. Surplus NADPH may be used to a greater extent by GR, allowing for a higher reduction by antioxidants involved in the Foyer–Halliwell–Asada cycle (directly, glutathione pool; and indirectly, ascorbate pool). Under ammonium nutrition, both low-mass antioxidant pools (ascorbate and glutathione) of *rbohd* plants were reduced more than in control plants (Figure 3 and Figure 4a). In this context, the downregulation of GR2 observed in situations of higher availability of NADPH in plant tissue in WT plants under ammonium stress was unexpected (Figure 4b and [52]), and even to a higher extent in *rbohd* plants grown under ammonium nutrition (Figure 4b). Since GR activity possesses the lowest activity among all enzymes included in the Foyer–Halliwell–Asada cycle [98], it may be recognized as its regulatory point. The downregulation of GR under reduction stress may be a type of metabolic regulation for preventing the over-reduction of the organelle glutathione pool. Moreover, intracellular antioxidant status is influenced by importing DHA and exporting AsA from the apoplast. In apoplast, regeneration of DHA most probably did not occur, and apoplastic ascorbate pool reduction was strictly dependent on cytosolic antioxidant systems. In *rbohd* plants, ammonium nutrition led to limited oxidation of the apoplastic ascorbate pool, influencing the symplastic ascorbate oxidation level (Figure 3).

Under control growth conditions (nitrate nutrition), RBOHD dysfunction led to slightly increased hydrogen peroxide (Figure 2a), along with decreased ascorbate (Figure 3) and glutathione pool (Figure 4a) reduction levels, but no oxidative injuries were observed simultaneously (Figure 2b,c). A previous study showed that under nonstress conditions, plants with RBOHD dysfunction showed no visible differences compared to WT in hydrogen peroxide content at the tissue level measured by DAB staining [99], but possessed enhanced apoplastic hydrogen peroxide content [100]. Our findings suggested a mechanism similar to that found in other studies, in which RBOHD was necessary to initiate an antioxidant defense against stresses such as salt stress, pathogen response, and high light [101,102,103]. Based on the correlation between negative regulation of signaling genes under ammonium nutrition (Figure 6) and intracellular antioxidant capacity, it is tempting to speculate whether this effect is related to the loss of RBOHD or downstream-disturbed oxidative communication. A connection between RBOHD-derived signaling routes and antioxidant enzyme activation is frequently speculated, but intermediates are not entirely deciphered [25,104].

Overall, increased intracellular ROS levels in ammonium-grown *rbohd* (Figure 2a) and increased lipid peroxidation (Figure 2b) suggested the onset of oxidative stress. With the reduced growth of *rbohd* (Figure 1a), plants showed a higher susceptibility to ammonium toxicity. These results indicated that RBOHD plays an important role in the gap between stress perception and plant adaptation.

## 5. Conclusions

Metabolic activation of RBOHs due to perceiving stress conditions (in this case, ammonium nourishment) or the dysfunction of RBOHD in transgenic plants directly affects the apoplastic ROS pool. RBOHD activity forms a bridge between stress perception and plant adaptation. In a way, this road leads from metabolic changes connected to ammonium assimilation in the cytosol to apoplastic ROS production via RBOHD activity. These ROS can trigger changes in apoplastic oxidation state or cell-wall integrity, which activate signaling in the cytosol again. Possibly, a signaling loop is formed to reduce ROS production in the apoplast, in which RBOHD has an antioxidant signaling function (Figure 7).

Therefore, the ammonium-specific apoplastic ROS burst was impeded in *rbohd,* which led to the downregulation of several signaling intermediates. We expect that the RBOHD-dependent apoplastic ROS burst may impact communication with the cell interior. We concluded that RBOHD is necessary for the perception and propagation of plant adaptation to ammonium stress, and its loss leads to plant sensitivity.

## Data Availability

Data is contained within the article and Supplementary Materials.

## Supplementary Materials

The following are available online at https://www.mdpi.com/article/10.3390/antiox11040703/s1. Figure S1: The protein level of RBOHD in plasma membranes. A whole blot is shown, including three biological replicates. Statistical differences calculated using one-way analysis of variance (ANOVA) with Tukey’s post hoc test are indicated at *p*-value ≤ 0.01 (double asterisks, **) and *p*-value ≤ 0.05 (single asterisk, *). Figure S2: Phosphorylated nucleotide concentration in leaves. Statistical differences calculated using one-way analysis of variance (ANOVA) with Tukey’s post hoc test are indicated at *p*-value ≤ 0.01 ** and *p*-value ≤ 0.05 *. Figure S3: The level of pectic highly methyl esterified homogalacturonan estimated using the indirect enzyme-linked immunosorbent assay method. Comparison tests between *rbohd* and WT plants were performed on the mean experimental conditions for the nitrate (light grey) or ammonium (dark grey) growth regime separately. Statistical differences calculated using one-way analysis of variance (ANOVA) with Tukey’s post hoc test are indicated at *p*-value ≤ 0.01 ** and *p*-value ≤ 0.05 *. Table S1: Primer sequences used for RT-qPCR assays. Reference [105] is cited in the Supplementary Materials.
