# Supplementary material for: Respiratory Burst Oxidase Homolog D as a Modulating Component of Oxidative Response under Ammonium Toxicity
Source: Antioxidants (Basel). 2022 Apr 2;11(4):703. doi: 10.3390/antiox11040703 (PMC9031508; doi:10.3390/antiox11040703)
Supplement: Supplementary file 1 [file antioxidants-11-00703-s001.zip › Suppl_Table_primers_Burian_et_al.pdf]

Table S1. Primer sequences used for RT-qPCR assays.

Gene	AGI identification		Primer sequence/reference
AIR12	AT3G07390	LP: TACCTCTTATCAAACACCAAACCACA	RP: AGCGAGACGAAACAAGCAACG
FER	AT3G51550	Podgórska et al. 2017 [55]	
GR1	AT3G24170	Podgórska et al. 2018a [50]	
GR2	AT3G54660	Podgórska et al. 2018a [50]	
MPK3	AT3G45640	Podgórska et al. 2017 [S_1]	
OZF1	AT2G19810	Podgórska et al. 2018b [12]	
PP2A	AT1G13320	Czechowski et al. 2005 [51]	
POX33	AT3G49110	Podgórska et al. 2018b [12]	
POX34	AT3G49120	Podgórska et al. 2018b [12]	
WAK1	AT1G21250	LP: CTTCTTGTAACCACCATCGGCTT	RP: CCCTGCTCCTGAGAGTCGTT

[12] Podgórska, A.; Ostaszewska-Bugajska, M.; Tarnowska, A.; Burian, M.; Borysiuk, K.; Gardeström, P.; Szal, B. Nitrogen Source Dependent Changes in Central Sugar Metabolism Maintain Cell Wall Assembly in Mitochondrial Complex I-Defective Frostbite1 and Secondarily Affect Programmed Cell Death. *Int. J. Mol. Sci.* **2018**, *19*, 2206, doi:10.3390/ijms19082206.

[50] Podgórska, A.; Ostaszewska-Bugajska, M.; Borysiuk, K.; Tarnowska, A.; Jakubiak, M.; Burian, M.; Rasmusson, A.; Szal, B. Suppression of External NADPH Dehydrogenase—NDB1 in Arabidopsis Thaliana Confers Improved Tolerance to Ammonium Toxicity via Efficient Glutathione/Redox Metabolism. *Int. J. Mol. Sci.* **2018**, *19*, 1412, doi:10.3390/ijms19051412.

[51] Czechowski, T.; Stitt, M.; Altmann, T.; Udvardi, M.K.; Scheible, W.-R. Genome-Wide Identification and Testing of Superior Reference Genes for Transcript Normalization in Arabidopsis. *Plant Physiol.* **2005**, *139*, 5–17, doi:10.1104/pp.105.063743.

[55] Podgórska, A.; Burian, M.; Gieczewska, K.; Ostaszewska-Bugajska, M.; Zebrowski, J.; Solecka, D.; Szal, B. Altered Cell Wall Plasticity Can Restrict Plant Growth under Ammonium Nutrition. *Front. Plant Sci.* **2017**, *8*, 1344, doi:10.3389/fpls.2017.01344.

[S_1] Podgórska, A.; Burian, M.; Rychter, A.M.; Rasmusson, A.G.; Szal, B. Short-Term Ammonium Supply Induces Cellular Defence to Prevent Oxidative Stress in *Arabidopsis* Leaves. *Physiol. Plant.* **2017**, *160*, 65–83, doi:10.1111/ppl.12538.
