# Supplementary material for: Respiratory Burst Oxidase Homolog D as a Modulating Component of Oxidative Response under Ammonium Toxicity
Source: Antioxidants (Basel). 2022 Apr 2;11(4):703. doi: 10.3390/antiox11040703 (PMC9031508; doi:10.3390/antiox11040703)
Supplement: Supplementary file 1 [file antioxidants-11-00703-s001.zip › Supplementary_Figures_Burian_et_al.pdf]

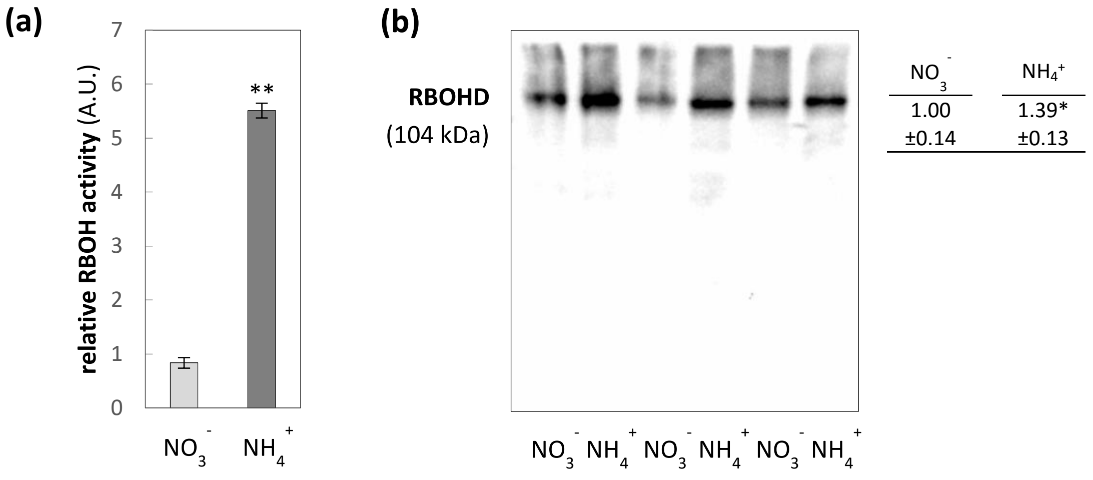


**Supplementary Figure S1**. RBOH activity and protein level in 8-week-old WT plants grown in hydroponic medium containing nitrogen in the form of nitrate or ammonium ions. (a) Relative activity of RBOH measured as DPI-sensitive superoxide production. (b) The protein level of RBOHD in plasma membranes. A whole blot is shown, including three biological replicates. Statistical differences calculated using one-way analysis of variance (ANOVA) with Tukey's posthoc test are indicated at p-value ≤0.01 (double asterisks, **) and p-value ≤0.05 (single asterisk,*).

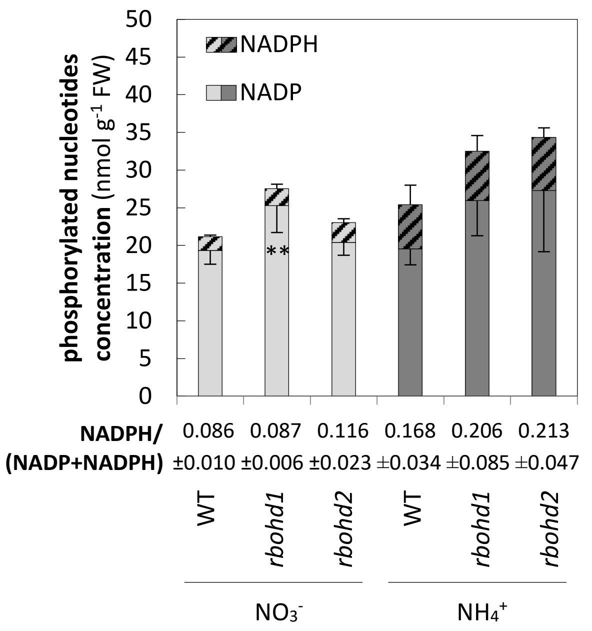


**Supplementary Figure S2**. Phosphorylated nucleotides concentration in leaves. Statistical differences calculated using one-way analysis of variance (ANOVA) with Tukey's posthoc test are indicated at p-value ≤0.01 **.

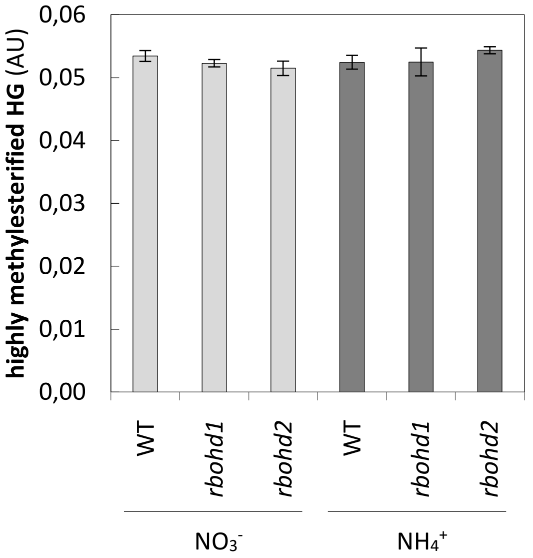


**Supplementary Figure S3**. The level of pectic highly methyl esterified homogalacturonan estimated using indirect enzyme-linked immunosorbent assay method. Comparison tests between *rbohd* and WT plants are performed on the mean experimental conditions for the nitrate (light grey) or ammonium (dark grey) growth regime separately.
